# Differences and Similarities for Spatial and Feature-Based Selective Attentional Orienting

**DOI:** 10.3389/fnins.2017.00283

**Published:** 2017-05-18

**Authors:** Daniela Galashan, Julia Siemann

**Affiliations:** ^1^Department of Neuropsychology and Behavioral Neurobiology, University of BremenBremen, Germany; ^2^Center for Advanced Imaging, University of BremenBremen, Germany

**Keywords:** feature-based attention, spatial attention, reorienting, cueing, fMRI, stimulus frequency

## Abstract

Using selective attention, we prioritize behaviorally relevant information out of all surrounding stimulation. Attention can be oriented intentionally to spatial and/or non-spatial properties (feature-based attention). When comparing spatial and feature-based attention, previous studies identified a common fronto-parietal network, although some reported specific activation for spatial attention and few found higher activation for feature-based attention. Most studies examining differences between attention types investigated the cueing epoch. We examined reorienting processing (after invalid cueing) and correctly focused attention (after valid cueing) for spatial and feature-based orienting using fMRI in two human samples with 40 participants overall and identical stimuli, stimulus probabilities, and timing for all conditions. A fronto-parietal network including parts of the ventral orienting network was activated for reorienting and focused attention for both attention types. Common activity over validities and attention types was located in bilateral IPL/SMG, bilateral IFG/insula, and the cerebellum. A network of mainly posterior areas showed higher activity for spatial compared to feature-based orienting. Conversely, no specialized areas for spatial focused attention or for feature-based attention (reorienting/focusing) was observed. The posterior clusters specialized for spatial reorienting showed overlapping activity with clusters involved in common spatial and feature-based reorienting as well as focused attention over attention types. Therefore, the results hint at a superordinate fronto-parietal network for both attention types during reorienting and focusing, with a spatial specialization of posterior sub-regions.

## Introduction

Being constantly confronted with multiple streams of information, we have to prioritize the most relevant input to be able act adequately and effectively. Through selective direction of attention we can focus intentionally on specific aspects of our environment, e.g., a certain position in space (spatial attention) or a specific feature like a particular color (feature-based attention). Cueing a location or feature correctly (valid cue; attentional focusing) leads to a performance improvement for a subsequently presented stimulus. In contrast, incorrect cueing (invalid cue) leads to a performance decline because reorienting is required to the unexpected target stimulus (Allport, 1971; Posner, 1980; Posner and Presti, 1987). This cueing effect (difference between valid and invalid trials) is also visible in attention-dependent activity modulations of neurons in early visual areas (Moran and Desimone, 1985; Treue and Maunsell, 1996; Roelfsema et al., 1998) as well as in increased activity modulations in early visual areas shown with fMRI (functional magnetic resonance imaging; O'Craven et al., 1999; Kastner and Ungerleider, 2000).

On a whole-brain level, attentional focusing usually activates a dorsal fronto-parietal network [including the frontal eye fields (FEF), superior parietal lobules (SPL), and intraparietal sulcus (IPS); see Hopfinger et al., 2000; Corbetta and Shulman, 2002]. In contrast, reorienting is commonly associated with activation in a right-lateralized ventral fronto-parietal network comprising the temporoparietal junction (TPJ) and ventral frontal areas (Corbetta and Shulman, 2002; Petersen and Posner, 2012). Nevertheless, an interaction between both networks seems to be evident (Vossel et al., 2014) and activation in both networks is reported for both reorienting and selective attention (see Kincade et al., 2005; Doricchi et al., 2010).

While the dorsal fronto-parietal network has mainly been found during spatial attention (Yantis et al., 2002), it also appears to be involved in feature-based attention (Liu et al., 2003). When comparing spatial and feature-based attention with fMRI, there are two main findings: First, concordant clusters in frontal (Giesbrecht et al., 2003; Egner et al., 2008) and parietal areas are reported (Wojciulik and Kanwisher, 1999; Vandenberghe et al., 2001; Greenberg et al., 2010). Second, concerning differences between spatial and non-spatial attention some studies reported areas with higher activation for spatial attention but no specific regions showing increased activation for feature-based attention (e.g., Egner et al., 2008). Nevertheless, there are also studies reporting increased activation during non-spatial compared to spatial cueing (Giesbrecht et al., 2003; Zanto et al., 2010). For example, Giesbrecht et al. (2003) found higher activation in posterior inferior temporal cortices and ventral extrastriate cortex for color cues compared to spatial cues. Additionally, a study by Greenberg et al. (2010) reported frontal and parietal areas to be involved in spatial as well as feature-based attention but with differing spatiotemporal activity patterns in posterior parietal cortex.

Most of those studies comparing spatial and non-spatial attention focused on the cueing period where attention had to be maintained on a specific feature (e.g., Vandenberghe et al., 2001; Giesbrecht et al., 2003; Slagter et al., 2007; Egner et al., 2008; Zanto et al., 2010). By contrast, brain activation during attentional reorienting (target presentation after invalid cueing), or focusing (target presentation after valid cueing) is less well-investigated. Those conditions can be compared to each other and to periods with no attentional orienting (target presentation on neutral trials) to characterize brain regions primarily involved in attentional reorienting or in focused attention *per-se*. Thus, it can be examined whether comparable brain regions process different types of attention (spatial/feature-based attention). Here, instead of focusing on the cue period like the majority of studies comparing spatial and feature-based attention, we focused on target presentation, allowing for an investigation of similarities during different attentional mechanisms like reorienting and focusing.

Many studies comparing spatial and feature-based attention did not use identical stimulation and timing between tasks, impeding proper comparisons between both tasks (see Carrasco, 2011 for a review; but see for example Greenberg et al., 2010). Furthermore, in cueing tasks invalid conditions usually have a lower probability than valid conditions, leading to differences in stimulus frequency between those conditions that are compared with each other. Indeed, the use of differing probabilities affects neuronal processing, as infrequent events violate participants' expectations (Kim, 2014 for a meta-analysis of the oddball effect). This expectation violation leads to increased reaction times RTs and distinct ERP components (Squires et al., 1975) and is also visible in altered activation obtained with imaging methods. For example, Vossel et al. (2006) found increased activation in a right fronto-parietal network when cue validity was increased from 60 to 90%. Furthermore, Corbetta and Shulman (2002) came to the conclusion that the detection of low-frequency events involves a ventral, mainly right-hemispheric fronto-parietal network comprising right TPJ and ventral frontal cortex [including inferior frontal gyrus (IFG), middle frontal gyrus (MFG), and frontal operculum]. As mentioned above, exactly these regions were also reported to be involved in attentional reorienting. Therefore, Macaluso and Doricchi, 2013) emphasized that it is important to distinguish possible effects caused by differing stimulus probabilities from attention effects evoked by selective attention and reorienting *per-se*. In the present studies we used equiprobable cueing conditions and the same timing and identical stimulation for both studies to examine reorienting independent of design parameters or violations of expectation. Consequently, cue reliability was rather low in this experiment (50% of the informative cues). Nevertheless, participants' compliance was achieved by stressing the need to follow the cue information and to direct attention according to the instruction. This approach has proven successful as reflected in the obtained behavioral data.

While past studies indeed investigated the influence of cue validity frequency with respect to spatial attention, none of these studies directly manipulated attention to features as an independent variable. The present results therefore add to existing knowledge about attentional mechanisms by introducing informative as well as uninformative (neutral) feature cues in order to examine whether feature cues would lead to comparable attention effects as spatial cues.

In the present studies, spatial and feature-based attention was manipulated in separate task designs using distinct samples to avoid several side effects. In particular, a design with intermixed spatial and feature-based trials would have provoked more universal task processing strategies eliciting activity in the fronto-parietal network (Shulman et al., 2002; Slagter et al., 2006; Stoppel et al., 2013). These effects would not be separable from attention effects in areas that are in the focus of the present study. Moreover, mixed designs have been shown to elicit a general switching mode causing shifting costs irrespective of the order of the tasks and largely unaffected by practice (Kleinsorge et al., 2004). Instead, in the present task participants were expected to be engaged in the corresponding task set (either spatial or feature-based attention) during the completion of the task. Finally, completing the same basic attention task first with spatial and then with feature-based attention (or vice versa) probably would produce learning effects that might be carried over from the first to the second type of orienting and thus influence the processing of the subsequently processed orienting type unpredictably.

Therefore, we used different participant samples for both tasks. Of course, this decision decreases the similarity between both data sets but in turn increases the possible generalization of concordant activations for both tasks because they constitute similarities over different participants.

Hence, our research question centered on the issue whether there is a common underlying mechanism for spatial and feature-based reorienting and focused attention (visible over distinct samples) when using tasks with comparable stimulation, timing, and stimulus probabilities. Furthermore, possible differences between spatial and feature-based processing during reorienting and focused attention were examined. Parts of the behavioral and fMRI data (spatial study) are also described elsewhere (see Siemann et al., 2016), with a focus on the results of the flanker task. Therefore, in the present paper the factor “flanker congruency” was ignored in the analyses of the MR data by pooling over congruent and incongruent flanker stimuli. The present manuscript can be considered as independent of the aforementioned publication due to other analyses, additional data (feature-based study) and a completely different focus leading to different conclusions. To our knowledge, no previous study used both, identical stimulation and timing between spatial and feature-based attention types while simultaneously examining cue validity with equal stimulus frequencies.

Based on findings in the literature, we hypothesized a graded attention effect of invalid > neutral > valid for RTs. On a neuronal level, we expected a common ventral fronto-parietal network to be involved in reorienting processing after invalid cueing for both tasks and mainly dorsal fronto-parietal areas to show common activities over tasks for focused attention after valid cueing. Concerning differences between both tasks we anticipated enhanced activity for spatial compared to feature-based attention in parts of the fronto-parietal network, whereas feature-based attention was expected to show only minor signal increases located in ventral visual or frontal areas when contrasted to spatial attention.

Summarized, we raised three issues. We wanted to:

1. Find areas activated for reorienting (invalid > neutral) common to both tasks (activity mainly expected in ventral fronto-parietal network),
2. Examine common regions activated by focused attention (valid > neutral) for both tasks (activity mainly expected in dorsal fronto-parietal areas), and
3. Assess differences between spatial and feature-based attention for reorienting as well as for focused attention (enhanced activity for spatial attention expected in fronto-parietal areas, enhanced activity for feature-based attention rather low, expected in ventral visual or frontal regions).

Briefly, we found mainly ventral, but also dorsal fronto-parietal structures to be involved for both attention types. Activities for reorienting as well as focused attention showed high similarities for both tasks despite the fact that different participant samples were used. Differences between tasks were only found for spatial vs. feature-based reorienting. These clusters were mainly located in posterior areas. Some of these clusters were also visible for reorienting and focused attention conjunct over both tasks. Therefore, similar structures seem to be involved in reorienting and focusing, with and a specialization for spatially directed reorienting for a subset of these regions.

## Experimental procedures

### Participants

Overall 41 healthy participants took part in the experiments, all with normal or corrected-to normal vision and no history of psychiatric or neurological disorders, or medication affecting the central nervous system or substance abuse. All participants were right-handed with a median laterality quotient of 100% (range = 77–100%) according to the Edinburgh Inventory by Oldfield (1971). Each volunteer attended three sessions (training session, and an EEG- or fMRI session in a counterbalanced order). The present study focuses on the fMRI sessions where participants completed a flanker task either with spatial or feature-based cueing (each individual only participated in one of the studies; spatial study: *n* = 20, mean age = 25.6 ± 4.7 years; feature-based study: *n* = 21, 24.9 ± 3.0 years, 10 male each). One data set of the spatial study had to be excluded due to low behavioral performance (mean error rate: 27.8%, standard deviation *SD*: 6.3; mean error rate averaged over all other participants: 4.0%, *SD*: 4.2).

All participants received either 30€ or course credit and gave informed and written consent before the measurements. The study protocol was designed and performed according to the Helsinki Declaration of the World Medial Association (Rickham, 1964) and approved by the local ethics committee of the University of Bremen.

### Stimuli and task

We introduced a Posner-like cueing task (Posner, 1980) presenting location or color cues (depending on spatial or feature-based attention task) combined with an Eriksen flanker task (Eriksen and Eriksen, 1974). The basic design and task procedure were identical between studies. For each study two flanker congruency levels (congruent/incongruent) were combined with three cueing validity levels (valid, neutral, invalid), resulting in six conditions presented with equal frequencies (congruency: 50%; overall validity: 33.33%). Informative cueing had a frequency of 50% as in half of all cases the cueing was correct (valid) and in the other half of trials the cueing was misleading (invalid). Neutral cueing was identical in both studies and uninformative concerning the location and color of the upcoming target stimulus. Congruency sequence effects (Gratton et al., 1992) were controlled for by using the same pseudo-randomized stimulus sequences for all participants where six single conditions followed each of the other six experimental conditions equally often throughout runs. Careful balancing of stimuli and experimental conditions prevented any confound between cue validity, attentional orienting, and stimulus presentation. All stimuli appeared equally often at each of the possible locations/in each of the colors to ensure that differences between compared conditions are only caused by the internal orienting of attention (or lack of orienting in the neutral condition). The same basic design with identical order of presentation of all stimuli ensured maximal comparability between tasks. The only difference in presented stimulation between tasks was the identity of the cue word. Stimuli were presented in five runs with short breaks in between. Each run consisted of 72 trials (12 per condition, resulting in 60 trials per condition).

A separate training session was completed before the experiments to familiarize with the task. Participants were instructed to follow the cued attribute (location or color) despite the relatively low frequency of valid cues. They were instructed to maintain fixation on the fixation point in the center of the screen throughout the experiment. In the spatial task volunteers were expected to orient attention covertly to the cued location, and in the feature-based task they were instructed to expect the target stimulus in the cued color.

We chose the letters “H” and “S” as target stimuli flanked by two either identical (congruent task condition) or different (incongruent task condition) letters on each side. Stimuli were presented above or below fixation (2.4° from fixation point to center of stimulus). Congruent flanker stimuli consisted of five identical letters (HHHHH, SSSSS; Arial, upper case, overall 8.45° × 1.88° and 7.8° × 1.93°, respectively) and incongruent flankers were flanked by the other letter (SSHSS, HHSHH; overall 8.01° × 1.93° and 8.28° × 1.93°). Participants responded with a button press to the identity of the central target letter (H or S) using the right index and middle fingers (counterbalanced across participants).

**Figure 1:**
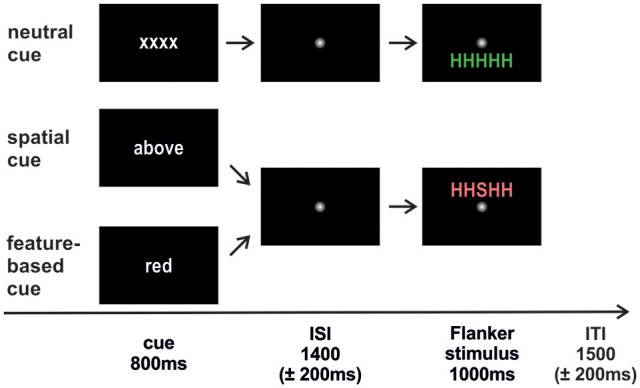
**Trial sequence**. The cue was followed by an interstimulus interval (ISI) where attention had to be directed to the cued feature. Afterwards the Flanker stimulus appeared and participants responded to the identity of the central target letter. An intertrial interval (ITI) with a fixation point separated the trials from each other. The neutral condition was identical in both studies. Spatial cueing indicated the to-be-attended stimulus position (above/below). Feature-based cueing indicated the to-be-attended stimulus color (red/green). Flanker stimuli could either be congruent (HHHHH; SSSSS) or incongruent (HHSHH; SSHSS) with equal probabilities.

The trial sequence is depicted in Figure 1. Each trial started with the presentation of a cue word in white font color on a black screen (800 ms; Arial, lower case). In the spatial task, the cue words “above” (“oben;” 3.39° × 1.25°) or “below” (“unten;” 3.75° × 1.2°) indicated a covert attention shift to the corresponding location. In the feature-based task, the cue words “green” (“grün;” 3.1° × 1.25°) and “red” (“rot;” 1.3° × 1.2°) were expected to focus attention on the respective color. The neutral cue “xxxx” (3.2° × 0.9°) was the same in both tasks. A smoothed fixation point (1.23° × 1.23°) was presented during the jittered interstimulus interval (ISI; 1,400 ± 200 ms) followed by the target letter with either congruent or incongruent flankers in red or in green above or below fixation (1,000 ms; 2.4° from fixation). Trials terminated with a smoothed fixation point presented for a jittered intertrial interval (ITI, 1,500 ± 200 ms).

### Data acquisition and data analysis

During the training session participants sat in a dimly lit room on a height adjustable chair with their heads on a chin and forehead rest in front of a 19-inch computer monitor (Belnea 1970 S1; distance 55 cm). For stimulus presentation we used Presentation® software (Neurobehavioral Systems; http://www.neurobs.com/). An in-house developed (MRI compatible) eye tracking device allowed monitoring of eye movements to ensure fixation on the central fixation point throughout the whole experiment.

A repeated-measures ANOVA was conducted on RTs using the within-subjects factors validity (valid, neutral, invalid) and Flanker congruency (congruent, incongruent) and the between-subjects factor task (spatial, feature-based attention). Paired as well as unpaired *t*-tests were used for *post-hoc* comparisons depending on the respective contrast. As the error rate data did not follow a normal distribution (according to Kolmogorov–Smirnov tests), non-parametric *U*-tests were conducted to compare data across studies.

### fMRI data

Functional and structural MRI data were obtained from a 3-T Siemens Skyra® whole body system equipped with a 20 channel head coil for image acquisition. Participants lay on a scanner couch inside the MR scanner and stimuli were projected via a mirror attached to the head coil (distance to projection area: 140 cm). Changes in blood oxygenation level-dependent (BOLD) T2^*^-weighted MR signals were measured by a gradient echo-planar imaging (EPI) sequence (TR = 2,210 ms, TE = 30 ms, flip-angle = 81°, matrix = 64^*^64, FOV = 192^*^192, voxel size = 27 mm^3^, 41 slices, no gap, ascending acquisition order, 163 volumes per run). Additionally, a T1-weighted structural image was acquired (MPRAGE; 176 slices; TR = 1,900 ms, TE = 2.07 ms, TI = 900 ms, voxel size = 1 mm^3^, flip angle = 9°). Image analysis was performed using the Statistical Parametric Mapping software package (SPM 8; Wellcome Department of Imaging Neuroscience, London, UK; http://www.fil.ion.ucl.ac.uk/spm/ software/). fMRI data were spatially realigned (10th volume of the first run) using a six parameter rigid-body transformation (4th degree B-Spline interpolation) and slice-time corrected to the middle slice in time. Structural MRI data were reoriented, segmented, and used for co-registration of the functional data. Both structural and functional images were normalized to the standard MNI space (4th degree B-Spline, including standard resampling to 2 mm^3^ isotropic voxels for the functional data). fMRI data were smoothed spatially (8 mm Gaussian kernel, full-width, half-maximum; FWHM), serial autocorrelations were corrected by an AR(1) model, and a standard high-pass filter of 128 s was used to remove low frequency drifts.

For further details on the SPM data analysis please see Siemann et al. (2016). The correct trials of all six experimental conditions, the cue period, and the ISI were used as separate regressors, being modeled as events using the canonical hemodynamic response function (Della-Maggiore et al., 2002) for each study (spatial/feature-based) and used for a fixed-effects analysis on individual data. The ISI was defined as a separate epoch because it refers to the interval between cue and stimulus with either spatial or feature-based attentional focusing. Therefore, it was not classified as a typical baseline epoch that might not be defined as an regressor. Six motion parameters (three translations and three rotations) were modeled separately as regressors of no interest to account for motion artifacts. Individual contrasts were included in multi-subject random effects analyses (Holmes and Friston, 1998) to account for inter-subject variance. The data were analyzed in a full factorial design on a second level using an ANOVA with the between-factor task (spatial/feature-based) and the within-subjects factors validity (valid, neutral, invalid) and Flanker congruency (congruent, incongruent). *Post-hoc* paired *t*-tests were performed on the reorienting contrast (invalid > neutral) separately for both tasks as well as on the focused attention contrast (valid > neutral) separately for both tasks to evaluate distinct effects for both tasks. These direct contrasts were carried out on a whole brain level using a family-wise-error correction (FWE) with a threshold of *p* ≤ 0.05 and a cluster extent threshold of *k* ≥ 20 voxels.

Afterwards, two conjunctions were computed to assess activity common to both tasks, one for the reorienting contrast, and one for the focused attention contrast. As the observed clusters were partly overlapping for reorienting and focused attention, a conjunction (conjunction null according to Nichols et al., 2005) was computed to determine brain areas showing activation in each of the four contrasts (common activity for spatial as well as for feature-based reorienting and for spatial and feature-based focused attention). The conjunction contrasts were carried out with a threshold of *p* ≤ 0.001 (uncorrected) and a cluster extent threshold of *k* ≥ 20 voxels. This more liberal threshold was chosen because these conjunctions constitute comparisons over two different data sets and we did not want to eliminate common clusters that might not have shown up with a rather strict correction. Moreover, this threshold is in conformity with the already published results on another aspect of the spatial study (Siemann et al., 2016).

To examine differences between attention types, unpaired *t*-tests with the two samples as independent groups were run on the reorienting contrast (invalid > neutral) for spatial > feature and also for feature > spatial tasks. The same procedure was also applied to the focused attention contrast (valid > neutral). All four contrasts were also computed *p* <0.001 (uncorrected, *k* ≥ 20 voxels) as they also constitute comparisons between different samples.

Anatomical assignments of activated regions were determined using the “Automated Anatomical Labeling” tool (AAL, Tzourio-Mazoyer et al., 2002), the Talairach Atlas (Talairach and Tournoux, 1988), and the “Talairach Daemon Client” software (http://www.talairach.org/daemon.html). For comparison with the Talairach space, peak coordinates of activated brain regions were converted using the MATLAB script “mni2tal.m” (http://imaging.mrc-cbu.cam.ac.uk/imaging/MniTalairach). To investigate whether the current clusters contain activation in the putative area FEF, the observed coordinates were compared to FEF coordinates provided by Paus (1996) and by Amiez and Petrides (2009). For that purpose the corresponding contrast was displayed in MRICron (Rorden and Brett, 2000) and it was checked whether the FEF coordinates provided by the above-mentioned publications coincided within the activated clusters. If this was the case the cluster was assumed to contain putative FEF activation. Overlaps between different contrasts were also confirmed with a visual inspection of both contrasts simultaneously in MRICron.

## Results

### Behavioral data

**Figure 2:**
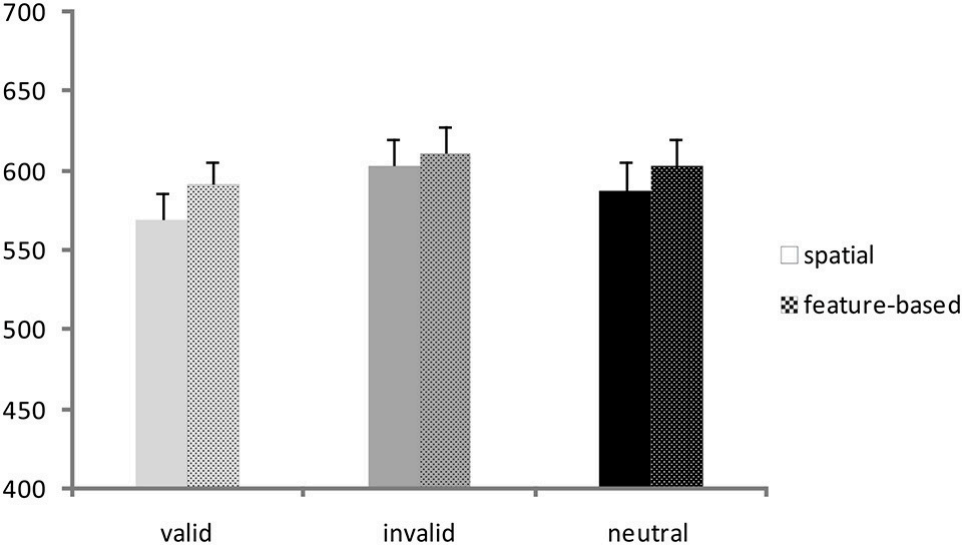
**Behavioral data**. Reaction times (ms) for valid, invalid, and neutral conditions in the spatial study (solid colored bars) and the feature-based study (patterned bars).

A repeated-measures ANOVA was conducted on RTs with validity (valid, invalid, neutral) and Flanker congruency (congruent, incongruent) as within-subjects factors and task (spatial, feature-based study) as between-subjects factor. Corresponding RT bar charts are depicted in Figure 2 and mean RT-values of all conditions are provided in Table 1 (left side). A significant main effect of validity [*F*_(2, 76)_ = 25.9; *p* <0.001] yielded lowest RTs for the valid condition and highest RTs for the invalid condition (mean ± *SD*: valid: 580 ± 70 ms; invalid: 607 ± 75 ms; mean neutral: 596 ± 74 ms). The flanker main effect was also significant [*F*_(1, 38)_ = 97.0; *p* <0.001] with higher RTs in the incongruent condition (incongruent: 610 ± 70 ms; congruent: 578 ± 75 ms). There were no other significant interactions or task effects [mean ± *SD*: spatial study: 587 ± 72 ms; feature-based study: 602 ± 72 ms; main effect of task: *F*_(1, 38)_ = 0.45; *p* = 0.51]. In order to examine differences between validity conditions, data from both studies were pooled and analyzed with paired *post-hoc t*-tests. The *t*-tests yielded significant differences between all validity conditions [valid vs. invalid: *t*_(39)_ = −6.8; *p* <0.001; invalid vs. neutral: *t*_(39)_ = 2.9; *p* = 0.007; valid vs. neutral: *t*_(39)_ = −4.3; *p* <0.001].

**Table 1:** **Behavioral data**.

		**RTs [ms]** ± ***SD***		**Error rates[%]** ± ***SD***	
	**Condition**	**Congruent**	**Incongruent**	**Congruent**	**Incongruent**
Spatial study *N* = 19	Valid	548.3 ± 70.8	583.3 ± 74.4	3.7 ± 4	5.5 ± 5.8
	Neutral	570.1 ± 75.8	605.6 ± 76.7	3.1 ± 3.8	4.2 ± 3.3
	Invalid	583.3 ± 74.4	622.6 ± 67.9	2.7 ± 2.9	4.6 ± 4.7
Feature-based study *N* = 21	Valid	577 ± 73.6	605.4 ± 63.8	3.7 ± 3	4.4 ± 2.8
	Neutral	591.1 ± 75.4	616.4 ± 71.3	3.8 ± 2.5	4.6 ± 3.3
	Invalid	598.7 ± 84.9	622.5 ± 75.8	8.3 ± 4.7	4.8 ± 3.9

*Summary of the mean reaction times (RTs, left) and percentage error rates (right) for the six conditions of the spatial study (top) and the feature-based study (bottom) with standard deviations (SD)*.

### Error rates

Percentage error rates were examined to ensure a comparable difficulty level across tasks. Mean error rates for all conditions are depicted in Table 1 (right side). As the data were not normally distributed (Kolmogorov–Smirnov test), non-parametric *U*-tests were conducted to compare the error rates of the six experimental conditions across tasks. Only the *U*-test for the comparison of the invalid congruent condition yielded a significant result (*Z* = −3.8; *p* <0.001), indicating higher error rates in this condition in the feature-based data compared to the spatial data.

### fMRI data

#### Reorienting

At first, activation for the reorienting contrast (invalid >neutral) with spatial attention is described, then the same contrast under feature-based attention, and afterwards the conjunction of both contrasts is presented to show areas involved in reorienting irrespective of attention type.

**Figure 3:**
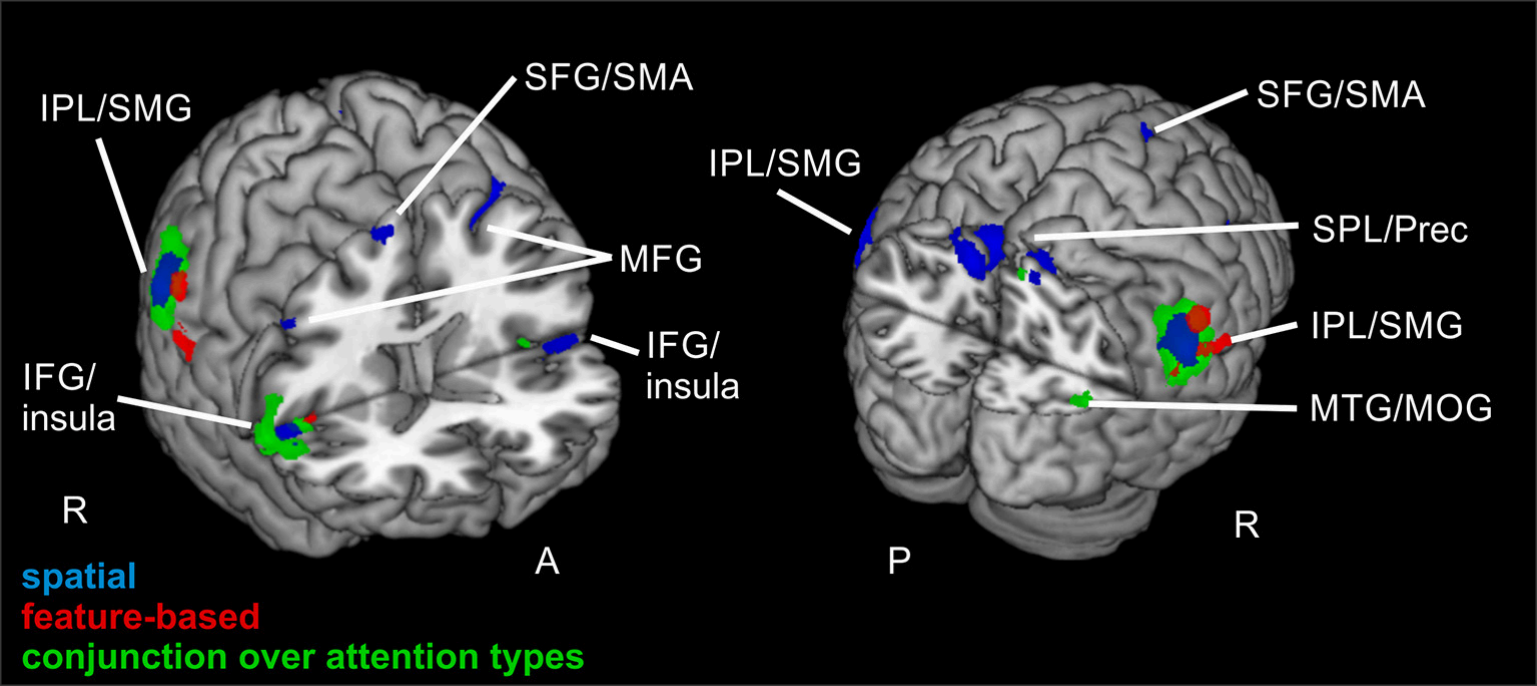
**Reorienting for spatial and feature-based attention**. Overlay of activated clusters for the reorienting contrast (invalid > neutral) are shown in blue for spatial attention, in red for feature-based attention (both with FWE-correction, *p* < 0.05, *k* ≥ 20) and the conjunction over both attention types is shown in green (uncorrected, *p* < 0.001, *k* ≥ 20). IFG, inferior frontal gyrus; IPL, inferior parietal lobule; MFG, middle frontal gyrus; MOG, middle occipital gyrus; MTG, middle temporal gyrus; Prec, precuneus; SFG, superior frontal gyrus; SMA, supplementary motor area; SMG, supramarginal gyrus; SPL, superior parietal lobule; STG, superior temporal gyrus; R, right hemisphere; A, anterior; P, posterior.

Contrasting invalid and neutral conditions for *spatial* attention (FWE-corrected, *p* < 0.05, *k* ≥ 20 voxels) yielded clusters in bilateral supramarginal gyrus SMG (left-hemispheric with a subpeak superior temporal, right-hemispheric with the main peak label in IPL). Further bilateral clusters were found in bilateral MFG and in bilateral SFG. The cluster in left MFG reflected involvement of putative area FEF and both clusters in SFG contained activity in left and right supplementary motor area SMA (according to the “AAL” tool, Tzourio-Mazoyer et al., 2002). Another set of bilateral clusters was visible in IFG. Although only the subpeak of the right-hemispheric cluster was located in IFG (main peak label: superior temporal gyrus STG), according to AAL both clusters might mostly contain activity in opercular parts of the IFG and in the insula. Two further clusters were located in left and right MFG. According to AAL, the right-hemispheric cluster might solely contain activity in pars orbitalis of the IFG. Another cluster was found with a peak label in left precuneus and a subpeak in SPL, spanning activity over of both hemispheres. The last cluster yielded a peak label in right superior occipital gyrus and a subpeak in right precuneus. According to AAL, the activity might be located mainly in MOG. Coordinates (MNI) for this contrast and the corresponding reorienting contrast for feature-based attention are listed in Table 2. Activated clusters are depicted in blue in Figure 3.

**Table 2:** **fMRI activations for reorienting separately for attention type**.

	**Reorienting (Spatial task)**						**Reorienting (Feature-based task)**					
**Anatomical region (BA)**	**Side**	**Cluster size**	***t*-value**	**Peak coordinate (MNI)**			**Side**	**Cluster size**	***t*-value**	**Peak coordinate (MNI)**		
SMG (BA 40)	L	831	8.17	−60	−48	28						
STG (BA22)			5.80	−46	−60	14						
MFG (BA 6)	L	453	7.50	−34	−2	58						
Precuneus (BA 7)	L	953	7.45	−10	−62	56						
SPL	R		6.78	14	−60	60						
IPL/SMG (BA 40)	R	421	7.31	64	−42	30	R	52	5.47	62	−32	36
IPL							R	40	5.40	56	−44	22
IPL							R	67	5.49	64	−26	22
SFG/SMA	R	140	7.03	12	14	64						
STG	R	235	6.92	48	12	0						
IFG (BA 47)			5.44	44	26	−6						
IFG/insula (BA47)	L	239	6.56	−48	14	0						
MFG	R	186	5.94	46	8	42						
			5.10	42	0	56						
SFG/SMA		82	5.40	0	16	52						
MFG (BA 47)	R	37	5.39	48	40	−6						
												
MFG (BA 10)	L	41	5.16	−32	44	20						
SOG /MOG	R	23	5.16	38	−76	24						
Precuneus			4.89	32	−76	32						
Cerebellum							L	384	5.76	−28	−66	−26
									5.59	−12	−64	−22
									5.18	−32	−60	−22
LG (BA 18)							R	67	5.58	6	−78	−2
Insula							R	47	5.36	40	6	2

*MNI coordinates of significant activation peaks for reorienting (invalid > neutral) for the spatial task (left column) and for the feature-based task (right column; both FWE-corrected, p < 0.05; k ≥ 20 voxels). Anatomical classifications of the clusters are listed on the left. BA, Brodmann Area; IFG, inferior frontal gyrus; IPL, inferior parietal lobule; L, left hemisphere; LG, lingual gyrus; MFG, middle frontal gyrus; R, right hemisphere; SFG, superior frontal gyrus; SMA, supplementary motor area; SMG, supramarginal gyrus; SOG, superior occipital gyrus; SPL, superior parietal lobule; STG, superior temporal gyrus*.

When looking at the same contrast (invalid >neutral, FWE-corrected, *p* < 0.05, *k* ≥ 20 voxels) for *feature-based* attention, a pattern with more inferior clusters was found. The broadest cluster was located in cerebellum. Further activation was visible in lingual gyrus spanning activity in both hemispheres. Another cluster with a peak label in postcentral gyrus yielded activity mainly in SMG (according to AAL). Two clusters with peaks in right IPL also showed activity in SMG. The last cluster was located in right insula (see Table 2 for corresponding coordinates and red clusters in Figure 3).

The conjunction over both attention types for reorienting (invalid > neutral, conjunction over spatial and feature-based tasks, uncorrected, *p* < 0.001, *k* ≥ 20 voxels) showed bilateral clusters in IPL/SMG as well as bilateral insular clusters. One further cluster was located in left cerebellum and another in right precuneus with a subpeak in MTG (activation mainly in MOG according to AAL). Another cluster was found in right MFG. The coordinates for the conjunction over attention types for reorienting, as well as the conjunction over attention types for focused attention are found in Table 3. Activated clusters are shown in green in Figure 3.

**Table 3:** **Common fMRI activations for reorienting and for focused attention over attention types**.

	**Reorienting (conjunction over attention types)**						**Focused attention (conjunction over attention types)**					
**Anatomical region (BA)**	**Side**	**Cluster size**	***t*-value**	**Peak coordinate (MNI)**			**Side**	**Cluster size**	***t*-value**	**Peak coordinate (MNI)**		
IPL (BA 40)/SMG	R	968	5.09	59	−33	35	R	676	5.47	57	−35	33
IPL (BA 40)			4.79	57	−39	26			4.17	57	−41	41
IPL (BA 40)			4.52	63	−34	22						
Insula (BA 13)/FIO/PCG (BA 44)	R	591	4.76	44	8	0	R	414	5	55	12	3
STG (BA 22)			4.36	55	12	1						
IFG (BA47)			4.05	51	19	1						
Declive/culmen	L	216	4.6	−32	−59	−19	L	350	4.89	−32	−57	−21
Declive			4.26	−40	−57	−22			3.49	−22	−69	−15
Precuneus (BA 19)/MOG	R	202	4.5	30	−74	31	R	23	3.82	40	−73	26
MTG (BA 39)			4.11	42	−73	24						
IPL (BA 40)/SMG	L	143	4.13	−59	−41	26	L	179	4.48	−57	−39	26
Precuneus (BA 7)	R	25	3.83	10	−61	58	R	29	3.79	12	−61	58
Insula	L	29	3.8	−42	6	0	L	75	3.97	−42	6	0
MFG (BA 10)/SFG	R	41	3.69	22	55	19	R	20	3.52	24	53	19
SPL (BA 7)/precuneus							L	29	4	−20	−59	60
MFG (BA 6)/SFG							R	70	3.99	30	1	61
Insula (BA 6)/SFG/PCG							L	29	3.93	−28	−3	63
SFG (BA 40)/SMG/PoCG							L	43	3.87	−53	−24	31
IPL (BA 6)/SMA/SFG							R	23	3.79	16	11	60
MTG (BA 19)/MOG							R	29	3.79	12	−61	58

*MNI coordinates of significant activation peaks for reorienting (invalid > neutral; left column) and for focused attention (valid > neutral; right column) conjunct over attention types (spatial; feature-based; p < 0.001; k ≥ 20 voxels). Anatomical classifications of the clusters are listed on the left. BA, Brodmann Area; FIO, frontal inferior operculum; IFG, inferior frontal gyrus; IPL, inferior parietal lobule; L, left hemisphere; MFG, middle frontal gyrus; MTG, middle temporal gyrus; PCG, precentral gyrus; PoCG, postcentral gyrus; R, right hemisphere; SFG, superior frontal gyrus; SFG, superior frontal gyrus; SPL, superior parietal lobule; STG, superior temporal gyrus*.

### Focused attention

Comparable to the activations for reorienting, activated clusters for focused attention are presented first for the spatial task, afterwards for the feature-based task and in the end a conjunction is described over both tasks to reveal common activation.

**Figure 4:**
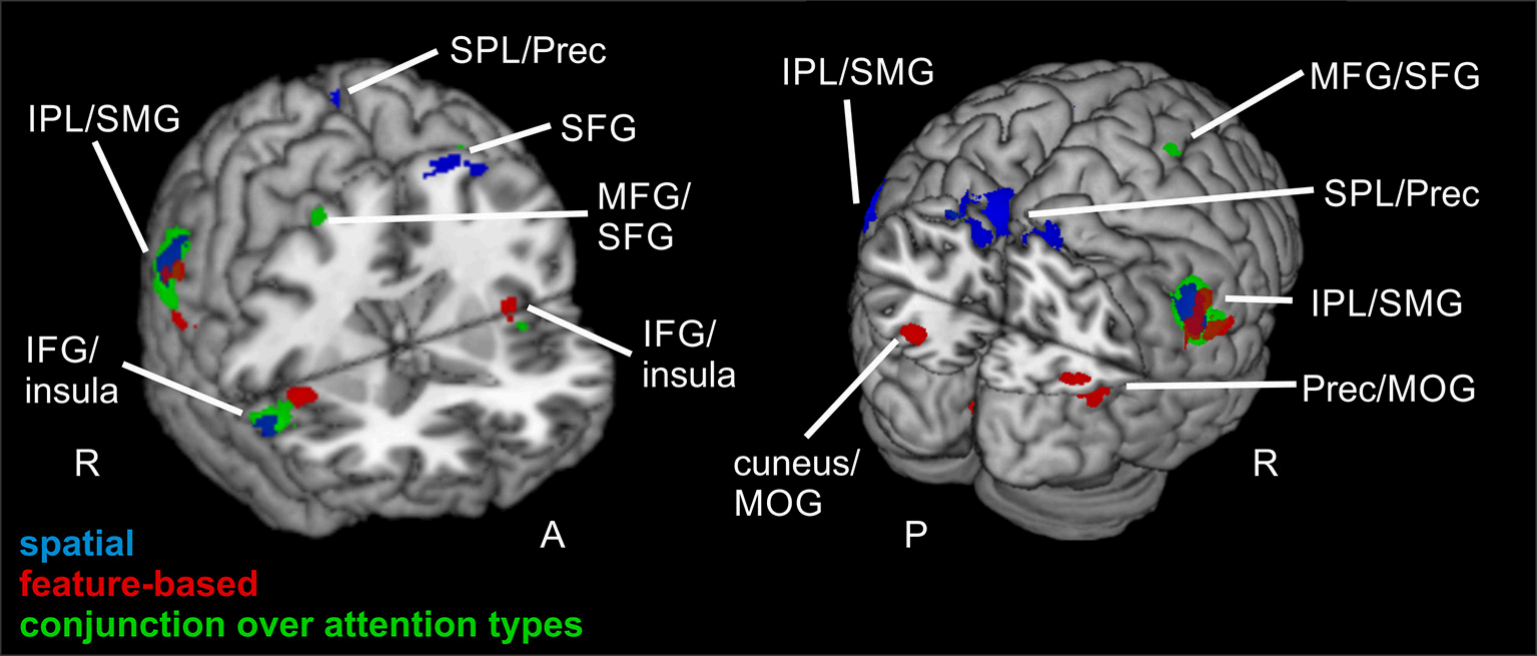
**Focused attention for spatial and feature-based attention**. Overlay of activated clusters for the focused attention contrast (valid > neutral) are shown in blue for spatial attention, in red for feature-based attention (both with FWE-correction, *p* < 0.05, *k* ≥ 20) and the conjunction over both attention types is shown in green (uncorrected, *p* < 0.001, *k* ≥ 20). IFG, inferior frontal gyrus; IPL, inferior parietal lobule; MFG, middle frontal gyrus; MOG, middle occipital gyrus; MTG, middle temporal gyrus; Prec, precuneus; SFG, superior frontal gyrus; SMG, supramarginal gyrus; SPL, superior parietal lobule; STG, superior temporal gyrus; R, right hemisphere; A, anterior; P, posterior.

The focused attention contrast (valid > neutral, FWE-corrected, *p* < 0.05, *k* ≥ 20 voxels) for *spatial* attention resulted in activity in bilateral SMG. Another posterior cluster was located in left precuneus (with subpeaks in left and right SPL). Further clusters were found in left SFG and right IFG (including activity in opercular parts of IFG). Corresponding coordinates for the focused attention contrast for spatial attention as well as feature-based attention are visible in Table 4 and clusters in blue in Figure 4.

**Table 4:** **fMRI activations for focused attention separately for attention types**.

	**Focused attention (spatial task)**						**Focused attention (feature-based task)**					
**Anatomical region (BA)**	**Side**	**Cluster size**	***t*-value**	**Peak coordinate (MNI)**			**Side**	**Cluster size**	***t*-value**	**Peak coordinate (MNI)**		
SMG (BA 40)/IPL (BA 40)	L	265	6.81	−57	−45	32	L	533	6.45	−57	−30	24
TTG (BA 42)										−57	−17	16
Insula (BA 13)										−42	−28	16
Precuneus (BA 7)	L	359	6.11	−8	−53	60						
SPL (BA 7)			5.42	14	−55	62						
SPL (BA)			5.14	−20	−59	60						
SMG/IPL (BA 40)	R	147	5.96	55	−37	37	R	673	6.35	61	−30	22
IPL (BA 40)			5.43	61	−37	30				59	−31	35
SMA (BA 40)										59	−39	30
SFG (BA 6)/PCG	L	70	5.4	−18	1	64						
MFG (BA 6)			5.29	−30	−1	57						
IFG (BA 45)/SMG/insula	R	37	5.34	57	14	1	R	193	6.22	40	6	2
STG (BA 22)										55	10	3
LG (BA 18)							R	3974	7.93	6	−76	0
Declive									6.97	−12	−65	−14
Declive									6.83	−24	−63	−17
Cuneus/MOG (BA 18)							L	270	7.07	−24	−81	21
Insula (BA 13)							L	156	6.24	−42	−4	6
Precuneus (B 19)							R	460	6.19	30	−74	31
MTG (BA 19)									6.11	38	−75	22

*MNI coordinates of significant activation peaks for focused attention (valid > neutral) for the spatial task (left column) and for the feature-based task (right column; both FWE-corrected, p < 0.05; k ≥ 20 voxels). Anatomical classifications of the clusters are listed on the left. BA, Brodmann Area; IFG, inferior frontal gyrus; IPL, inferior parietal lobule; L, left hemisphere; LG, lingual gyrus; MFG, middle frontal gyrus; MTG, middle temporal gyrus; PCG, precental gyrus; R, right hemisphere; SFG, superior frontal gyrus; SMA, supplementary motor area; SMG, supramarginal gyrus; SPL, superior parietal lobule; STG, superior temporal gyrus; TTG, transverse temporal gyrus*.

When examining focused attention (valid > neutral, FWE-corrected, *p* < 0.05, *k* ≥ 20 voxels) for *feature-based* attention, a large cluster was visible in right lingual gyrus, spanning activation in bilateral fusiform and lingual gyri, as well as large portions of the cluster in the cerebellum (according to AAL). The cluster with the main peak label in left cuneus might contain mainly activity in MOG (according to AAL). Bilateral clusters were found in IPL/SMG including activity in STG, as well as in insula (including activation in opercular parts of IFG). Another cluster with the main peak label in precuneus and a subpeak in MTG seemed to represent mainly activity in right MOG (see corresponding coordinates for this contrast in Table 4 and red clusters in Figure 4).

The conjunction contrast for focused attention (valid > neutral, conjunction over spatial and feature-based tasks, uncorrected, *p* < 0.001, *k* ≥ 20 voxels) yielded a large cluster in right IPL/SMG, as well as two smaller clusters in left IPL/SMG that might be regarded as bilateral to the right-hemispheric cluster. Further activation was visible in cerebellum and a different cluster with a peak label in right precentral gyrus (including activation in opercular parts of IFG and in right insula). Left insula also showed activation. Two posterior clusters might be regarded as bilateral although their peak labels differ: One was located in left SPL and the other in right precuneus. According to AAL, both clusters might mainly include activity in SPL as well as precuneus. Three further clusters showed activity in right MFG, one of them including activity in right SMA according to AAL. Two bilateral clusters with peak labels in MTG seem to contain almost exclusively activation in MOG (see coordinates in Table 3 and green clusters in Figure 4).

### Common activity for reorienting and focused attention over tasks

**Figure 5:**
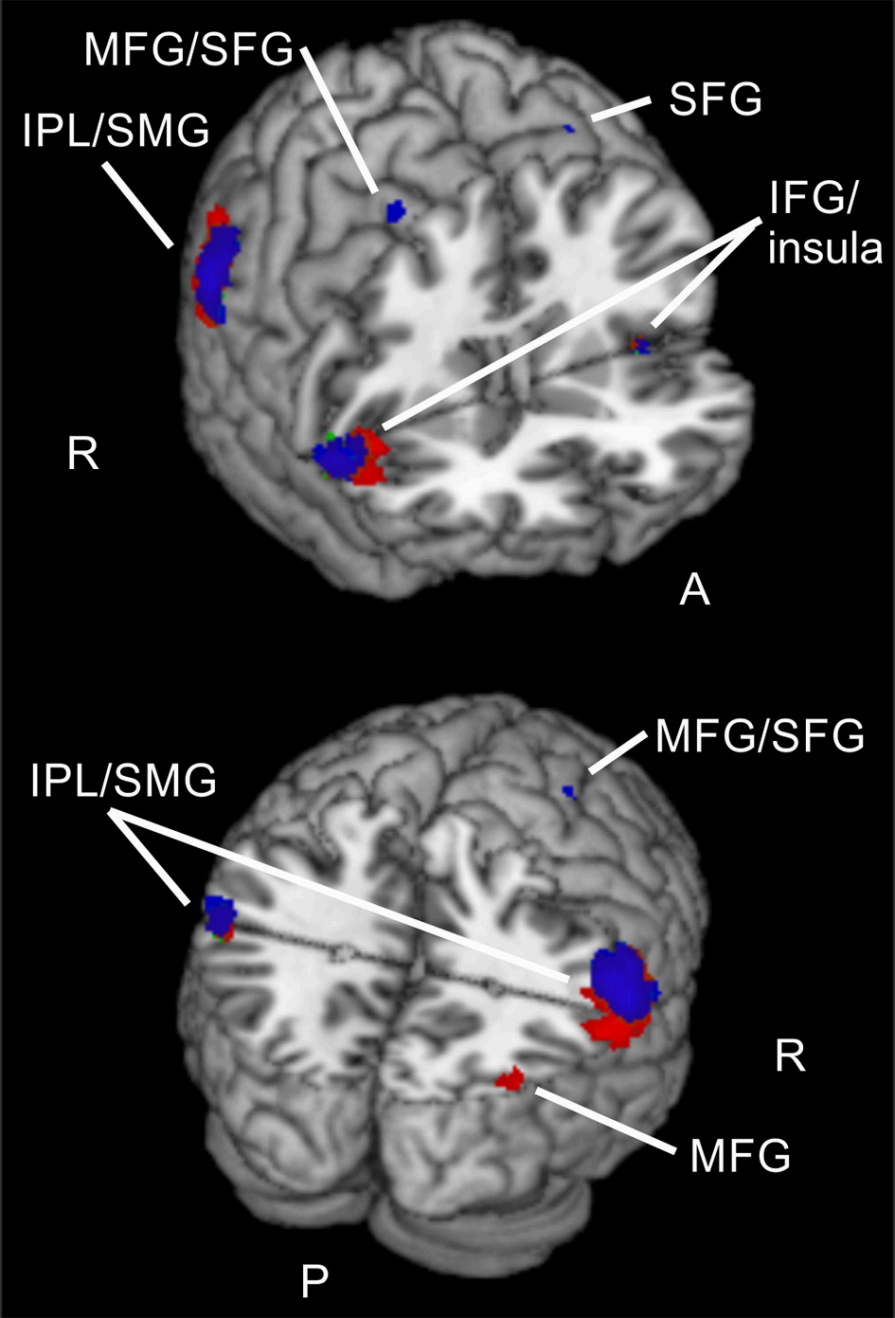
**Common activations for different validities and attention types**. Overlay of activated clusters for reorienting over attention types (conjunction) in red and focused attention over attention types (conjunction) in blue (both uncorrected, *p* < 0.001, *k* ≥ 20). IFG, inferior frontal gyrus; IPL, inferior parietal lobule; MFG, middle frontal gyrus; SFG, superior frontal gyrus; SMG, supramarginal gyrus; R, right hemisphere; A, anterior; P, posterior.

**Figure 6:**
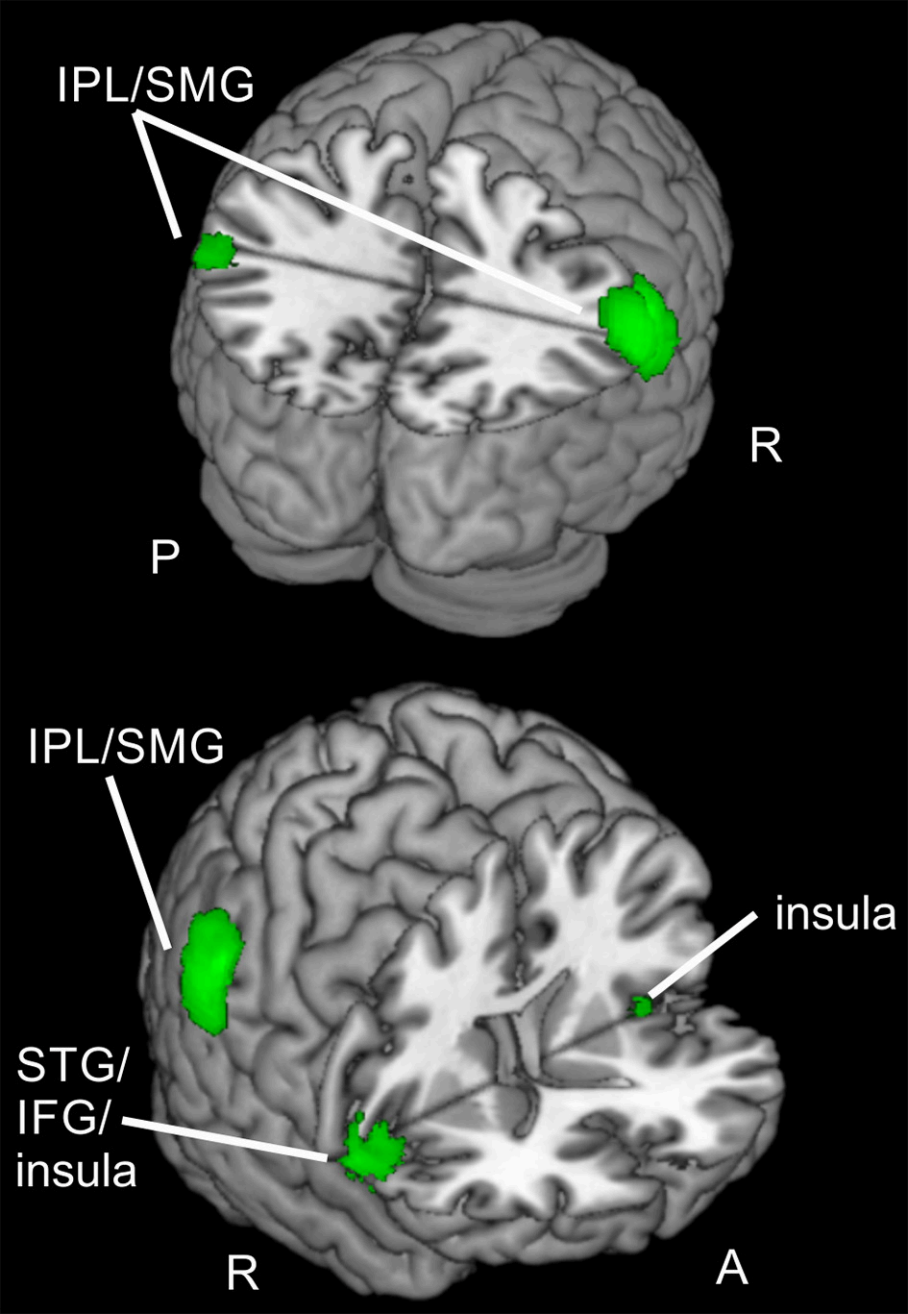
**Regions active for spatial and feature-based reorienting and focusing**. Overlay of activated clusters for reorienting over attention types (conjunction) in red and focused attention over attention types (conjunction) in blue (both uncorrected, *p* < 0.001, *k* ≥ 20). IFG, inferior frontal gyrus; IPL, inferior parietal lobule; SMG, supramarginal gyrus; STG, superior temporal gyrus; R, right hemisphere; A, anterior; P, posterior.

As activated clusters for reorienting and focused attention showed several comparable clusters (see Figure 5), we computed a conjunction null (uncorrected, *p* < 0.001, *k* ≥ 20 voxels) showing only clusters that are activated in each of the single contrasts. This conjunction null yielded similarities for reorienting and focused attention under spatial and feature-based attention (used contrasts: “invalid > neutral for spatial attention,” “invalid > neutral for feature-based attention,” “valid > neutral for spatial attention,” “valid > neutral for feature-based attention”). The conjunction resulted in bilateral clusters in IPL/SMG, another cluster in left cerebellum, and almost bilateral clusters with activity in insular cortex. The peak label of the right-hemispheric cluster was located in STG, but according to AAL it might correspond mainly to inferior frontal (pars opercularis, rolandic operculum) and insular cortex. The coordinates for this contrast are listed in Table 5 and clusters are shown in Figure 6.

**Table 5:** **Common fMRI activations for reorienting and focused attention over attention types**.

	**Attention (reorienting/focused; conjunction over attention types)**					
**Anatomical region (BA)**	**Side**	**Cluster size**	***t*-value**	**Peak coordinate (MNI)**		
IPL (BA 40)/SMG	R	582	4.95	59	−33	37
SMG (BA 40)			4.77	59	−39	30
IPL (BA 40)			4.1	57	−41	41
Declive	L	200	4.6	−32	−59	−19
STG (BA 22/FIO	R	344	4.36	55	12	1
STG (BA 22)	R		4.03	48	12	−2
IPL (BA 40)/SMG	L	131	4.13	−59	−41	26
Insula	L	29	3.8	−42	6	0

*MNI coordinates of the activation peaks for the conjunction null over 4 contrasts including spatial/feature-based reorienting (invalid > neutral) as well as spatial/feature-based focused attention (valid > neutral) (p < 0.001; k ≥ 20 voxels). Anatomical classifications of the clusters are listed on the left. BA, Brodmann Area; FIO, frontal inferior operculum; IPL, inferior parietal lobule; L, left hemisphere; R, right hemisphere; SMG, supramarginal gyrus; STG, superior temporal gyrus*.

### Differences between attention types

#### Reorienting

**Figure 7:**
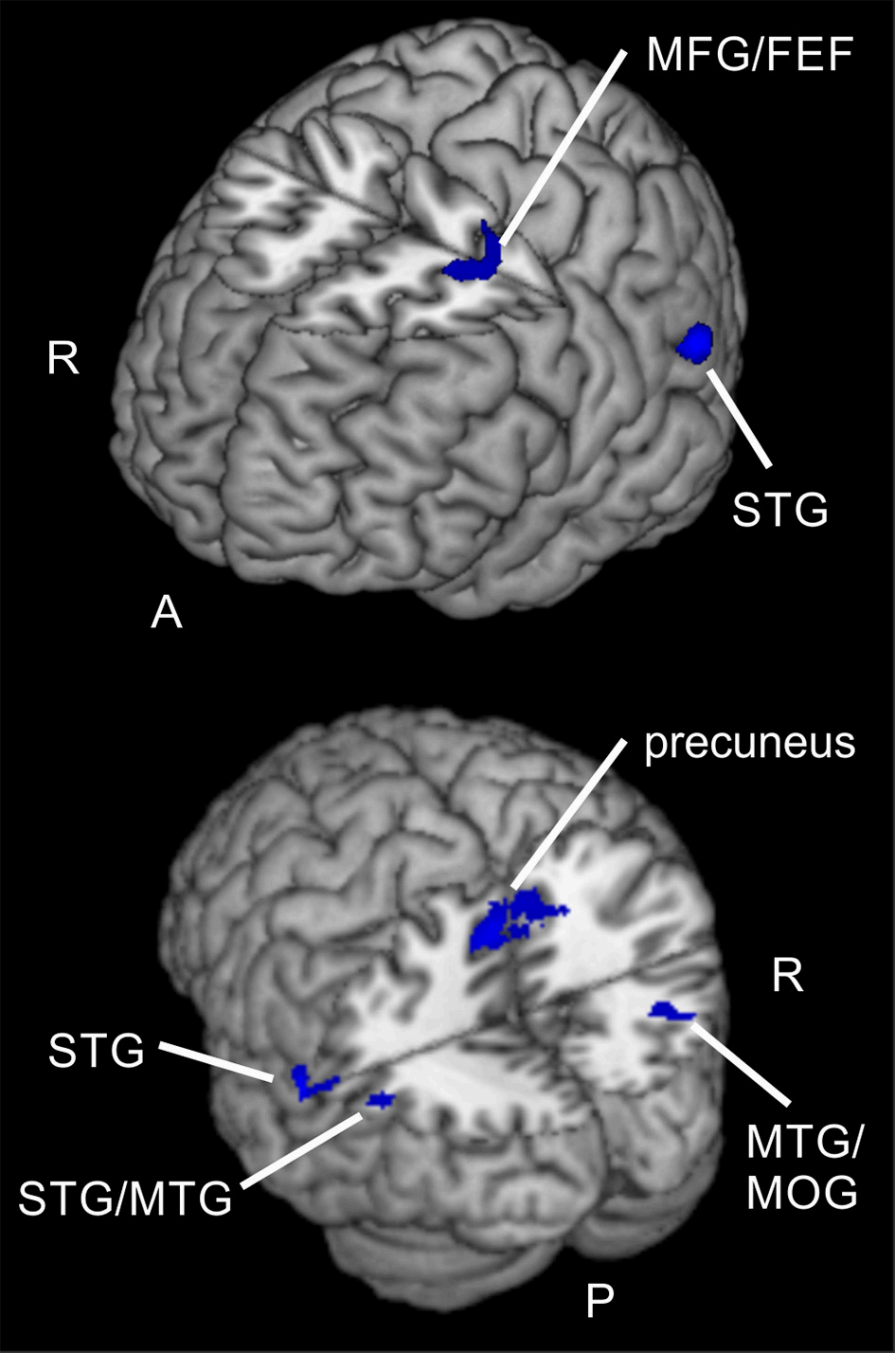
**Spatial compared to feature-based reorienting**. Overlay of activated clusters for a *t*-test showing higher activity for spatial compared to feature-based attention in the reorienting contrast (invalid > neutral, uncorrected, *p* < 0.05, *k* ≥ 20). FEF, frontal eye field; MFG, middle frontal gyrus; MOG, middle occipital gyrus; MTG, middle temporal gyrus; STG, superior temporal gyrus; R, right hemisphere; A, anterior; P, posterior.

When examining differences between attention types for reorienting (invalid > valid) with a two-sample *t*-test (uncorrected, *p* < 0.001, *k* ≥ 20 voxels), we observed one cluster spanning bilateral precuneus (BA 7), another cluster in left STG (BA 22) and a cluster in right middle temporal gyrus MTG (BA 39) that might include mainly activity in MOG (according to AAL). Another cluster was located in left STG (BA 39) and might contain mainly activity in MTG (according to AAL). A cluster in left parietal lobe was classified as “sub-gyral” and the only frontal cluster was observed in left MFG (BA 6). This cluster reflected involvement of putative area FEF. The corresponding coordinates for these clusters can be found in Table 6 and clusters are shown in Figure 7. The opposite contrast (invalid > valid for feature> spatial) did not produce any activated clusters with the given threshold (uncorrected, *p* < 0.001, *k* ≥ 20 voxels).

**Table 6:** **fMRI activations for reorienting with spatial compared to feature-based attention**.

	**Spatial attention**>**feature-based attention (reorienting)**					
**Anatomical region (BA)**	**Side**	**Cluster size**	***t*-value**	**Peak coordinate (MNI)**		
Precuneus (BA 7)	L	968	5.26	−10	−55	56
Precuneus (BA 7)	L		4.87	−8	−48	50
Precuneus (BA 7)	R		4.84	10	−54	51
STG (BA 22)	L	102	5.2	−61	−44	19
MTG (BA 39)	R	222	4.59	40	−73	26
STG (BA 39)	L	101	4.39	−46	−59	18
Sub−gyral (BA 40)	L	27	4.18	−34	−41	33
MFG (BA6)	L	74	3.79	−28	0	46
MFG (BA 6)	L		3.58	−32	−1	53

*MNI coordinates of significant activation peaks for spatial compared to feature-based attention for the reorienting contrast (invalid > neutral) (p < 0.001; k ≥ 20 voxels). Anatomical classifications of the clusters are listed on the left. BA, Brodmann Area; L, left hemisphere; MFG, middle frontal gyrus; MTG, middle temporal gyrus; R, right hemisphere; STG, superior temporal gyrus*.

#### Focused attention

For the focused attention contrast (valid > neutral) there were also no visible clusters with the given threshold, neither for the spatial > feature two-sample *t*-test, nor for the opposite *t*-test (feature > spatial, each uncorrected, *p* < 0.001, *k* ≥ 20 voxels).

As similarities between these clusters and the already described conjunctions for reorienting and focused attention over tasks were obvious, we checked these contrasts for overlapping clusters. Visually comparing the clusters in MRICron (Rorden and Brett, 2000) confirmed different overlaps. The conjunction for reorienting over attention types showed activity in left IPL/SMG overlapping with activity from the left STG cluster for spatial > feature-based attention during reorienting. Furthermore, there was overlapping activity in right precuneus for both contrasts and a large overlap in right MTG/MOG.

When looking at the conjunction for focused attention over attention types, overlaps with the contrast showing higher activity for spatial > feature-based attention during reorienting were observed in left IPL/SMG with the same STG cluster for spatial > feature-based attention during reorienting that was described before (as overlapping with the conjunction for reorienting). Two areas with overlaps were found in left and right precuneus, near the both peaks of the large precuneus cluster fron the spatial vs. feature-based attention contrast for reorienting. Another large overlap was located in right MTG/MOG.

Summarized, brain areas in left IPL/SMG, bilateral precuneus, and right MTG/MOG seem to be involved in reorienting and focused attention for spatial and for feature-based attention and these regions additionally do show higher activity for spatial attention when compared to feature-based attention during reorienting.

## Discussion

The reaction time analysis yielded main effects of validity and flanker as expected, confirming that the experimental design with equal probabilities for all conditions proved successful. Percentage error rates confirmed similar difficulty levels for both tasks, only the invalid congruent condition revealed higher error rates for feature-based data compared to spatial data. Thus, for five of six experimental conditions a comparable task difficulty in both tasks was ensured, indicating that differences in activation between both data sets might not originate from differing levels of difficulty.

In the following we will initially discuss brain activity separately and common to both attention types (spatial, feature-based) for reorienting and then for focused attention. Thereafter, we will focus on common activations across task condition (reorienting, focused attention) and attention types. In the end, we will discuss differences between attention types.

Reorienting (invalid > neutral) for spatial attention was associated with signal increases in structures of the dorsal fronto-parietal network (see Corbetta and Shulman, 2002), including MFG/FEF, SFG (including activity in SMA), precuneus/SPL, as well as in parts of the ventral orienting network (bilateral insula, bilateral IPL/SMG). This pattern of results showed a good correspondence to the clusters reported by Doricchi et al. (2010) for their comparison of invalid vs. neutral conditions in a spatial cueing task (e.g., activity in right MFG, bilateral precuneus/SPL, bilateral insula, right IFG, and bilateral TPJ). Reorienting for feature-based attention yielded a pattern with more ventral clusters (bilateral lingual gyrus, IPL/SMG, right insula, and cerebellum) and mostly activity in the ventral orienting network. These clusters were not so well in correspondence with the activations described by Doricchi et al. (2010). This is probably the case because they used a spatial rather than feature-based cueing task.

Common activated regions for spatial and feature-based reorienting consisted almost exclusively of parts of the ventral orienting network (bilateral IPL/SMG, bilateral insula), but one cluster was located in right MFG and another with a peak in right precuneus but presumably mainly activity in MOG. This pattern confirms our first hypothesis that parts of the ventral orienting network are involved in reorienting processing for both attention types. Nevertheless, also parts of the dorsal fronto-parietal network were engaged in reorienting, mainly for the spatial task.

The focused attention contrast (valid >neutral) also showed a more dorsal activation pattern for the spatial task and more ventral clusters for feature-based attention, comparable to the contrasts for reorienting. For focused attention in the spatial task parts of the dorsal fronto-parietal network were activated (left precuneus/SPL, left SFG). Activity in the ventral orienting network was also observed, in bilateral SMG and right IFG. Focused attention for feature-based attention yielded activity in lingual/fusiform gyri (and cerebellum), left MOG, bilateral IPL/SMG, bilateral insula, and right precuneus/MTG/MOG. When compared to the clusters described by Doricchi et al. (2010) for their “valid > neutral” contrast, the cluster in left SFG found in our spatial attention task resembled their cluster in left FEF and the cluster in left IPL/SMG was located close to their cluster in left TPJ. For our feature-based task, only the cluster in left insula resembled the location of their cluster in left IFG. As already mentioned, they used a spatial task, this might explain that only one cluster of our feature-based task was roughly comparable to their activation.

Despite the more ventral activity pattern of the feature-based task for focused attention, the conjunction (focused attention over attention types) also showed activity in left SPL and right precuneus, as well as three clusters in right MFG (one including activity in right SMA). Further clusters were found in the ventral orienting network: in bilateral IPL/SMG, left insula and right IFG/insula. Clusters in bilateral MTG/MOG and the cerebellum were also visible. Thus, our hypothesis to observe mainly activity in the dorsal fronto-parietal network for focused attention was not confirmed as there was more activity in regions assigned to the ventral orienting network. We have to note that we used equal stimulus probabilities to prevent any influences of different probabilities on neuronal processing. This might have influenced the results and reduced the comparability to other studies.

When looking at the clusters for reorienting and focused attention, a similarity in activated brain regions is obvious. The conjunction null, yielding only regions active for reorienting as well as focused attention in both tasks, showed clusters in bilateral IPL/SMG, bilateral IFG/insula, and left cerebellum. Doricchi et al. (2010) report overlapping activity in left TPJ for invalid compared to neutral and valid vs. neutral conditions. In our conjunction over both comparisons (invalid > neutral, valid > neutral) and both tasks we observed bilateral clusters in IPL/SMG. Other studies examining different attention types also reported overlapping activation in fronto-parietal areas (e.g., FEF, pre-SMA, IFG and anterior insula, bilateral IPS, and SPL; Giesbrecht et al., 2003; Egner et al., 2008; Greenberg et al., 2010). For example, in a conjunction analysis over spatial and feature-based cue information, Egner et al. (2008) observed activity e.g., in bilateral IFG/anterior insula, bilateral IPS, FEF, and left pre-SMA/ACC. Another study (Greenberg et al., 2010) reported overlapping activity for location and color processing in response to shift vs. hold cues in bilateral medial/SFG, left MFG/IFG, left middle occipital gyrus MOG, and bilateral medial SPL/precuneus. However, these studies found activity in several regions of the dorsal fronto-parietal network whereas the areas activated by spatial as well as feature-based attention in our tasks were mainly located in the ventral orienting network. Nevertheless, areas of the ventral orienting network also found in their pattern of results for the cueing epoch are in good correspondence with the present areas activated for reorienting and focused attention when integrating data over both tasks (bilateral IFG/insula, bilateral IPL). Most other studies (e.g., Egner et al., 2008) examined the cue period. Compared to these findings, it might be concluded that these parts of the network are probably activated jointly for spatial and non-spatial attention irrespective of task phase. This might lead to the conclusion that regions in bilateral IFG/insula and bilateral IPL are only involved during spatial as well as feature-based attention but also during different task phases, namely target presentation (including reorienting with invalid cueing and focused attention with valid cueing) and attentional build-up during the cueing period (as shown by other studies).

Summarized, two bilateral regions of the ventral orienting network (IPL/SMG, IFG/insula), as well as the cerebellum were involved when reorienting and focused attention were processed in both tasks. Thus, the activated clusters for reorienting and attentional focusing during spatial and feature-based attention in the present data suggest that the observed ventral fronto-parietal regions are not specialized for reorienting processes but more generally involved in spatial as well as feature-based directed attention with or without reorienting. These regions might even be involved in attentional processing irrespective of task phase as we found them for the target epoch whereas other authors (e.g., Egner et al., 2008) describe their activity during the cueing epoch.

Regarding differences between tasks, only the comparison for reorienting showed increased activity for the spatial task compared to the feature-based task. Clusters were located in bilateral precuneus, left STG/MTG, right MTG/MOG, parietal lobe and in putative FEF (BA 6). Compared to results of former studies, these areas are in good correspondence for comparisons between different types of attention. For example, Giesbrecht et al. (2003) found activity in right precuneus, left MTG, right IPL, left SPL, and left SFG for location vs. color conditions under peripheral stimulation. These clusters were located in the neighborhood of our clusters in precuneus, left STG, right MTG, parietal lobe and left putative FEF. Another study (Greenberg et al., 2010) also reported activity in bilateral precuneus and right MOG whereas Egner et al. (2008) observed increased activity for spatial cue information in right precuneus and left MFG (BA 6). Indeed, in contrast to our results the last-mentioned observation did not result from direct contrasts between spatial and feature-based attention conditions.

Summarized, our pattern of activation for spatial compared to feature-based reorienting corresponds to regions already reported to show differences between attention types.

For focused attention no suprathreshold clusters yielded higher activity for spatial compared to feature-based attention. However, this assures that there were no simple baseline differences between both participant groups that might have caused differences across all conditions and thus lead to the afore-mentioned differences for reorienting. Apparently, differences between attention types are more pronounced for reorienting than for focused attention. Either there are no actual differences between spatial and feature-based focused attention, the differences were to subtle to detect with the present tasks/data sets, or there are interindividual differences between participants that might have impeded the detection of common differences over participants.

Furthermore, with the given threshold we did not observe specific activation patterns for feature-based compared to spatial attention, neither for reorienting, nor for focused attention. This finding is consistent with data from previous studies comparing both types of attention (see Egner et al., 2008) where no specific network for feature-based attention could be observed.

Interestingly, there was overlapping activity when comparing these clusters showing higher activity for spatial reorienting to the clusters obtained in the conjunctions over tasks for reorienting and for focused attention. These overlaps were located in left IPL/SMG, bilateral precuneus, and right MTG/MOG. This means that these regions are activated in reorienting and focused attention during both tasks but also show an even higher activation when spatial reorienting is performed compared to feature-based reorienting. Indeed, apart from the right MTG/MOG activations, the other clusters showed rather small overlaps. We therefore speculate that different subparts within these regions might be specialized in a different way: on the one hand for selective attention (valid and invalid compared to neutral conditions) and on the other hand for spatial reorienting (compared to feature-based reorienting). These results are in accordance with results from Greenberg et al. (2010) who reported common domain-independent signals in posterior parietal and prefrontal cortex for spatial and non-spatial attention shifts but differing spatiotemporal activity patterns in posterior parietal cortex (but not in frontal areas) that were interpreted as reflecting domain-specificity. The finding of enhanced activity levels when dealing with spatial tasks compared to feature-based attention is corroborated by findings in the literature (Yantis et al., 2002; Giesbrecht et al., 2003; Stoppel et al., 2007). As mentioned before, different subregions of these areas might be specialized for one or another aspect.

## Conclusion

In summary, the expected pattern in the behavioral data (invalid > neutral > valid RTs) confirmed that attentional orienting was performed as requested despite equal stimulus probabilities for all conditions. Task difficulty was comparable for both tasks and both tasks were conducted with identical stimulation. A fronto-parietal network reported to be involved in attention tasks and including parts of the ventral orienting network was found to be activated for reorienting as well as focused attention for spatial and feature-based attention. The observed fronto-parietal network might be involved in different task epochs, i.e., target processing as in the present study or cueing epoch as analyzed in many other studies examining common spatial and feature-based activation. When directly assessing common activation for reorienting and focused attention of both tasks, clusters in bilateral IPL/SMG, bilateral IFG/insula, and cerebellum were detected.

Higher activity for spatial compared to feature-based reorienting was visible mainly in posterior areas (bilateral precuneus, left STG/MTG, right MTG/MOG, parietal lobe, FEF). For focused attention no task differences were found. Similarly, feature-based attention compared to spatial attention showed no task-specific network, neither for focused attention nor for reorienting. Interestingly, posterior clusters in left IPL/SMG, bilateral precuneus, and right MTG/MOG were active for reorienting and focused attention over task types and additionally showed increased activity during spatial compared to feature-based reorienting. Potentially, different subregions of the first-mentioned both regions might be involved in different aspects of task processing (reorienting, focused attention) and specialized for feature-based reorienting.

## Author contributions

DG conceived and supervised the studies. DG and JS designed the experiments. JS collected the data. JS and DG analyzed and interpreted the data. DG wrote the preliminary draft. JS and DG revised the manuscript. All authors read and approved the final manuscript.

### Conflict of interest statement

The authors declare that the research was conducted in the absence of any commercial or financial relationships that could be construed as a potential conflict of interest.

## Abbreviations

ACC: Anterior cingulate cortex
BA: Brodmann area
EEG: electroencephalography
FEF: Frontal eye field
fMRI: Functional magnetic resonance imaging
IFG: Inferior frontal gyrus
ISI: interstimulus interval
ITI: intertrial interval
IPL: Inferior parietal lobule
IPS: Intraparietal sulcus
MFG: Middle frontal gyrus
MOG: Middle occipital gyrus
MTG: Middle temporal gyrus
RTs: Reaction times
SD: Standard deviation
SFG: Superior frontal gyrus
SMA: Supplementary motor area
SMG: Supramarginal gyrus
SPL: Superior parietal lobule
STG: Superior temporal gyrus
TPJ: Temporoparietal junction.

## References

[B1] Allport D. A. (1971). Parallel encoding within and between elementary stimulus dimensions. Percept. Psychophys. 10, 104–108. 10.3758/BF03214327

[B2] Amiez C. Petrides M. (2009). Anatomical organization of the eye fields in the human and non-human primate frontal cortex. Prog. Neurobiol. 89, 220–230. 10.1016/j.pneurobio.2009.07.010 19665515

[B3] Carrasco M. (2011). Visual attention: the past 25 years. Vis. Res. 51, 1484–1525. 10.1016/j.visres.2011.04.012 21549742 PMC3390154

[B4] Corbetta M. Shulman G. L. (2002). Control of goal-directed and stimulus-driven attention in the brain. Nat. Rev. Neurosci. 3, 201–215. 10.1038/nrn755 11994752

[B5] Della-Maggiore V. Chau W. Peres-Neto P. R. McIntosh A. R. (2002). An empirical comparison of SPM preprocessing parameters to the analysis of fMRI data. Neuroimage 17, 19–28. 10.1006/nimg.2002.1113 12482065

[B6] Doricchi F. Macci E. Silvetti M. Macaluso E. (2010). Neural correlates of the spatial and expectancy components of endogenous and stimulus-driven orienting of attention in the Posner task. Cereb. Cortex 20, 1574–1585. 10.1093/cercor/bhp215 19846472

[B7] Egner T. Monti J. M. Trittschuh E. H. Wieneke C. A. Hirsch J. Mesulam M. M. (2008). Neural integration of top-down spatial and feature-based information in visual search. J. Neurosci. 28, 6141–6151. 10.1523/JNEUROSCI.1262-08.2008 18550756 PMC6670545

[B8] Eriksen B. A. Eriksen C. W. (1974). Effects of noise letters upon the identification of a target letter in a nonsearch task. Percept. Psychophys. 16, 143–149. 10.3758/BF03203267

[B9] Giesbrecht B. Woldorff M. G. Song A. W. Mangun G. R. (2003). Neural mechanisms of top-down control during spatial and feature attention. Neuroimage 19, 496–512. 10.1016/S1053-8119(03)00162-9 12880783

[B10] Gratton G. Coles M. G. Donchin E. (1992). Optimizing the use of information: strategic control of activation of responses. J. Exp. Psychol. Gen. 121, 480–506. 10.1037/0096-3445.121.4.480 1431740

[B11] Greenberg A. S. Esterman M. Wilson D. Serences J. T. Yantis S. (2010). Control of spatial and feature-based attention in frontoparietal cortex. J. Neurosci. 30, 14330–14339. 10.1523/JNEUROSCI.4248-09.2010 20980588 PMC3307052

[B12] Holmes A. P. Friston K. J. (1998). Generalisability, random effects and population inference. Neuroimage 7:S754.

[B13] Hopfinger J. B. Buonocore M. H. Mangun G. R. (2000). The neural mechanisms of top-down attentional control. Nat. Neurosci. 3, 284–291. 10.1038/72999 10700262

[B14] Kastner S. Ungerleider L. G. (2000). Mechanisms of visual attention in the human cortex. Annu. Rev. Neurosci. 23, 315–341. 10.1146/annurev.neuro.23.1.315 10845067

[B15] Kim H. (2014). Involvement of the dorsal and ventral attention networks in oddball stimulus processing: a meta-analysis. Hum. Brain Mapp. 3, 2265–2284. 10.1002/hbm.22326 PMC6868981 23900833

[B16] Kincade J. M. Abrams R. A. Astafiev S. V. Shulman G. L. Corbetta M. (2005). An event-related functional magnetic resonance imaging study of voluntary and stimulus-driven orienting of attention. J. Neurosci. 25, 4593–4604. 10.1523/JNEUROSCI.0236-05.2005 15872107 PMC6725019

[B17] Kleinsorge T. Heuer H. Schmidtke V. (2004). Assembling a task space: global determination of local shift costs. Psychol. Res. 68, 31–40. 10.1007/s00426-003-0134-9 12774231

[B18] Liu T. Slotnick S. D. Serences J. T. Yantis S. (2003). Cortical mechanisms of feature-based attentional control. Cereb. Cortex 13, 1334–1343. 10.1093/cercor/bhg080 14615298

[B19] Macaluso E. Doricchi F. (2013). Attention and predictions: control of spatial attention beyond the endogenous-exogenous dichotomy. Front. Hum. Neurosci. 7:685. 10.3389/fnhum.2013.00685 24155707 PMC3800774

[B20] Moran J. Desimone R. (1985). Selective attention gates visual processing in the extrastriate cortex. Science 229, 782–784. 10.1126/science.4023713 4023713

[B21] Nichols T. Brett M. Andersson J. Wager T. Poline J. B. (2005). Valid conjunction inference with the minimum statistic. Neuroimage 25, 653–660. 10.1016/j.neuroimage.2004.12.005 15808966

[B22] O'Craven K. M. Downing P. E. Kanwisher N. (1999). fMRI evidence for objects as the units of attentional selection. Nature 401, 584–587. 10.1038/44134 10524624

[B23] Oldfield R. C. (1971). The assessment and analysis of handedness: the Edinburgh inventory. Neuropsychologia 9, 97–113. 10.1016/0028-3932(71)90067-4 5146491

[B24] Paus T. (1996). Location and function of the human frontal eye-field: a selective review. Neuropsychologia 34, 475–483. 10.1016/0028-3932(95)00134-4 8736560

[B25] Petersen S. E. Posner M. I. (2012). The attention system of the human brain: 20 years after. Annu. Rev. Neurosci. 35, 73–89. 10.1146/annurev-neuro-062111-150525 22524787 PMC3413263

[B26] Posner M. I. (1980). Orienting of attention. Q. J. Exp. Psychol. 32, 3–25. 10.1080/00335558008248231 7367577

[B27] Posner M. I. Presti D. E. (1987). Selective attention and cognitive control. Trends Neurosci. 10, 12–17. 10.1016/0166-2236(87)90116-0

[B28] Rickham P. P. (1964). Human experimentation. Code of ethics of the world medical association. Declaration of Helsinki. Br. Med. J. 2:177. 10.1136/bmj.2.5402.177 14150898 PMC1816102

[B29] Roelfsema P. R. Lamme V. A. Spekreijse H. (1998). Object-based attention in the primary visual cortex of the macaque monkey. Nature 395, 376–381. 10.1038/26475 9759726

[B30] Rorden C. Brett M. (2000). Stereotaxic display of brain lesions. Behav. Neurol. 12, 191–200. 10.1155/2000/421719 11568431

[B31] Shulman G. L. D'Avossa G. Tansy A. P. Corbetta M. (2002). Two attentional processes in the parietal lobe. Cereb. Cortex 12, 1124–1131. 10.1093/cercor/12.11.1124 12379601

[B32] Siemann J. Herrmann M. Galashan D. (2016). fMRI-constrained source analysis reveals early top-down modulations of interference processing using a flanker task. Neuroimage, 136, 45–56. 10.1016/j.neuroimage.2016.05.036 27181762

[B33] Slagter H. A. Giesbrecht B. Kok A. Weissman D. H. Kenemans J. L. Woldorff M. G.. (2007). fMRI evidence for both generalized and specialized components of attentional control. Brain Res. 1177, 90–102. 10.1016/j.brainres.2007.07.097 17916338 PMC2710450

[B34] Slagter H. A. Weissman D. H. Giesbrecht B. Kenemans J. L. Mangun G. R. Kok A.. (2006). Brain regions activated by endogenous preparatory set shifting as revealed by fMRI. Cogn. Affect. Behav. Neurosci. 6, 175–189. 10.3758/CABN.6.3.175 17243354

[B35] Squires N. K. Squires K. C. Hillyard S. A. (1975). Two varieties of long-latency positive waves evoked by unpredictable auditory stimuli in man. Electroencephalogr. Clin. Neurophysiol. 38, 387–401. 10.1016/0013-4694(75)90263-1 46819

[B36] Stoppel C. M. Boehler C. N. Sabelhaus C. Heinze H. J. Hopf J. M. Schoenfeld M. A. (2007). Neural mechanisms of spatial- and feature-based attention: a quantitative analysis. Brain Res. 1181, 51–60. 10.1016/j.brainres.2007.07.019 17961522

[B37] Stoppel C. M. Boehler C. N. Strumpf H. Krebs R. M. Heinze H. J. Hopf J. M.. (2013). Distinct representations of attentional control during voluntary and stimulus-driven shifts across objects and locations. Cereb. Cortex 23, 1351–1361. 10.1093/cercor/bhs116 22593242

[B38] Talairach J. Tournoux P. (1988). Co-Planar Stereotaxic Atlas of the Human Brain. Stuttgart: Thieme.

[B39] Treue S. Maunsell J. H. (1996). Attentional modulation of visual motion processing in cortical areas MT and MST. Nature 382, 539–541. 10.1038/382539a0 8700227

[B40] Tzourio-Mazoyer N. Landeau B. Papathanassiou D. Crivello F. Etard O. Delcroix N. (2002). Automated anatomical labeling of activations in SPM using a macroscopic anatomical parcellation of the MNI MRI single-subject brain. Neuroimage 15, 273–289. 10.1006/nimg.2001.0978 11771995

[B41] Vandenberghe R. Gitelman D. R. Parrish T. B. Mesulam M. M. (2001). Location- or feature-based targeting of peripheral attention. Neuroimage 14, 37–47. 10.1006/nimg.2001.0790 11525335

[B42] Vossel S. Geng J. J. Fink G. R. (2014). Dorsal and ventral attention systems: distinct neural circuits but collaborative roles. Neuroscientist 20, 150–159. 10.1177/1073858413494269 23835449 PMC4107817

[B43] Vossel S. Thiel C. M. Fink G. R. (2006). Cue validity modulates the neural correlates of covert endogenous orienting of attention in parietal and frontal cortex. Neuroimage 32, 1257–1264. 10.1016/j.neuroimage.2006.05.019 16846742

[B44] Wojciulik E. Kanwisher N. (1999). The generality of parietal involvement in visual attention. Neuron 23, 747–764. 10.1016/S0896-6273(01)80033-7 10482241

[B45] Yantis S. Schwarzbach J. Serences J. T. Carlson R. L. Steinmetz M. A. Pekar J. J.. (2002). Transient neural activity in human parietal cortex during spatial attention shifts. Nat. Neurosci. 5, 995–1002. 10.1038/nn921 12219097

[B46] Zanto T. P. Rubens M. T. Bollinger J. Gazzaley A. (2010). Top-down modulation of visual feature processing: the role of the inferior frontal junction. Neuroimage 53, 736–745. 10.1016/j.neuroimage.2010.06.012 20600999 PMC2930130

